# Bovine viral diarrhoea virus seroprevalence and vaccination usage in dairy and beef herds in the Republic of Ireland

**DOI:** 10.1186/2046-0481-65-16

**Published:** 2012-07-31

**Authors:** D J Bosco Cowley, Tracy A Clegg, Michael L Doherty, Simon J More

**Affiliations:** 1 MSD Animal Health, Red Oak North, South County Business Park, Leopardstown, Dublin 18, Ireland; 2 School of Agriculture, Food Science and Veterinary Medicine, University College Dublin, Belfield, Dublin 4, Ireland; 3 Centre of Veterinary Epidemiology and Risk Analysis, School of Agriculture, Food Science and Veterinary Medicine, University College Dublin, Belfield, Dublin 4, Ireland

## Abstract

**Background:**

Bovine viral diarrhoea (BVD) is an infectious disease of cattle with a worldwide distribution. Herd-level prevalence varies among European Union (EU) member states, and prevalence information facilitates decision-making and monitoring of progress in control and eradication programmes. The primary objective of the present study was to address significant knowledge gaps regarding herd BVD seroprevalence (based on pooled sera) and control on Irish farms, including vaccine usage.

**Methods:**

Preliminary validation of an indirect BVD antibody ELISA test (Svanova, Biotech AB, Uppsala, Sweden) using pooled sera was a novel and important aspect of the present study. Serum pools were constructed from serum samples of known seropositivity and pools were analysed using the same test in laboratory replicates. The output from this indirect ELISA was expressed as a percentage positivity (PP) value. Results were used to guide selection of a proposed cut-off (PCO) PP. This indirect ELISA was applied to randomly constructed within-herd serum pools, in a cross-sectional study of a stratified random sample of 1,171 Irish dairy and beef cow herds in 2009, for which vaccination status was determined by telephone survey. The herd-level prevalence of BVD in Ireland (percentage positive herds) was estimated in non-vaccinating herds, where herds were classified positive when herd pool result exceeded PCO PP. Vaccinated herds were excluded because of the potential impact of vaccination on herd classification status. Comparison of herd-level classification was conducted in a subset of 111 non-vaccinating dairy herds using the same ELISA on bulk milk tank (BMT) samples. Associations between possible risk factors (herd size (quartiles)) and herd-level prevalence were determined using chi-squared analysis.

**Results:**

Receiver Operating Characteristics Analysis of replicate results in the preliminary validation study yielded an optimal cut-off PP (Proposed Cut-off percentage positivity - PCO PP) of 7.58%. This PCO PP gave a relative sensitivity (Se) and specificity (Sp) of 98.57% and 100% respectively, relative to the use of the ELISA on individual sera, and was chosen as the optimal cut-off since it resulted in maximization of the prevalence independent Youden’s Index.

The herd-level BVD prevalence in non-vaccinating herds was 98.7% (95% CI - 98.3-99.5%) in the cross-sectional study with no significant difference between dairy and beef herds (98.3% vs 98.8%, respectively, p = 0.595).

An agreement of 95.4% was found on Kappa analysis of herd serological classification when bulk milk and serum pool results were compared in non-vaccinating herds. 19.2 percent of farmers used BVDV vaccine; 81% of vaccinated herds were dairy. A significant association was found between seroprevalence (quartiles) and herd size (quartiles) (p < 0.01), though no association was found between herd size (quartiles) and herd-level classification based on PCO (p = 0.548).

**Conclusions:**

The results from this study indicate that the true herd-level seroprevalence to Bovine Virus Diarrhoea (BVD) virus in Ireland is approaching 100%. The results of the present study will assist with national policy development, particularly with respect to the national BVD eradication programme which commenced recently.

## Background

Bovine viral diarrhoea (BVD) is an infectious disease of cattle with a worldwide distribution [1]. In all countries where data are available, herd-level antibody prevalence to BVDV has averaged 55% [2]. An extensive review of the epidemiology and of the economic importance of BVDV is provided by Houe [3]. Transient infections in seronegative immunocompetent cattle in most cases are subclinical or result in mild signs including a transient fever and leucopenia. Occasionally, young animals may suffer from severe disease due to the immunosuppressive effect of the virus resulting in super-infections by opportunistic pathogens. The outcome of BVDV infection during pregnancy depends on the age of the fetus, and may result in foetal resorption, abortion, mummification, congenital malformations, birth of immunotolerant persistently infected and viraemic calves or birth of normal, weak or undersized calves [4]. Persistently infected (PI) calves shed large quantities of virus while having no or low levels of BVDV antibodies and can remain undetected in a herd or evolve to a highly fatal clinical illness known as “Mucosal Disease” [5]. PIs are the main source of viral transmission within herds [6], and trade of PIs or non-PI dams carrying PI fetuses constitutes the major route for the transmission of virus between herds [7]. Within-herd seroprevalence had varied between 19 and 89% [8,9] while the prevalence of PI animals in the entire cattle population ranges from 0.5% to 2% [3,10-13]. A number of European countries are either in advanced stages of eradication of BVD (Norway, Sweden, Denmark, Austria, Switzerland, Shetland (part of the U.K.)) or implemented regional control programmes (France, Germany, The Netherlands, Italy, U.K.). The control and eradication of BVD infections has been reviewed previously [14,15].

In Ireland, some information has recently emerged on BVD infection, albeit from a biased subset of Irish cattle [16], indicating a herd-level prevalence of 94%. Individual animal seroprevalence was found to vary annually between 64-69% over a sampling period of four years. Seropositivity was significantly higher in adults compared to juvenile stock. However, no data are available in Ireland on the strategies used to control infection, including vaccination. Significantly, as part of the national Animal Health Ireland (AHI) initiative, a recent Delphi study of experts and farmers identified BVD as among the most important animal health issues facing Irish livestock farmers, in terms of costs to farms and agribusiness [17]. Information regarding prevalence and existing control measures facilitates decision-making and monitoring of progress in control and eradication programmes.

The primary objective of the present study was to describe BVD herd-level seroprevalence and vaccine usage on dairy and beef farms in the Republic of Ireland. Preliminary validation of an indirect BVD antibody ELISA (Svanova; Biotech AB, Uppsala, Sweden) using pooled sera was conducted as part of this study.

## Methods

### Preliminary validation

Five hundred negative and 500 positive sera (‘the archived sera’) were selected from routine submissions to the diagnostic unit of Agri-Food and Biosciences Institute (AFBI) in Belfast. The archived sera were assayed using an indirect ELISA for BVDV antibodies (Svanova; Biotech AB, Uppsala, Sweden). These sera were classified into either of two groups – known BVDV antibody test positives or test negatives. A validation pool was classified positive if it contained ≥1 positive archived serum sample. The test was performed according to the instructions of the manufacturer. Both positive and negative control sera were included in each assay. Sensitivity (Se) and specificity (Sp) of the test when used on individual sera relative to serum neutralisation test (SNT) are 100% and 98.2%, respectively (Svanova, Data on file). Only values for Se and Sp of the indirect ELISA for BVDV antibody, when the test is used on individual serum samples, are available (and not pool Se (PSe) and pool Sp (PSp)) (Manufacturer data on file). We assume PSe = Se and PSp = Sp.

The archived sera were used to form a series of validation pools, each containing 30 sera (20 μL each, 600 μL for each sample pool). Specifically, each validation pool included a defined number (‘*n*’) of positive sera (where *n* = 0, 1, 2, 3, 4, 5, 6, 7, 8, 9, 10, 15, 20, 25 or 30) in combination with 30-*n* negative samples. For example, one validation pool had 0 positive and 30 negative samples, another had 1 positive and 29 negative samples, etc. In total, 90 validation pools were created, including 20 pools where n = 0, and 5 each for the 14 remaining positive/negative combinations. For each of these validation pools, the positive and negative samples were each selected using simple random sampling, using a computer generated random number list (Microsoft Excel 2003, Redmond, WA, USA), from the 500 known negative and 500 known positive sera. The 90 validation pools were analysed using the above-mentioned BVDV antibody ELISA, all at AFBI’s diagnostic unit. The absorbance or optical density (OD) of each well at 450 nm was measured on a microplate plate reader. The corrected optical density (COD) value of each pool and reference serum was obtained by subtraction of the OD value of each control antigen-coated well from that of the parallel viral antigen-coated well [18]. A corresponding percentage positivity (PP) value was obtained using the formula: PP value = COD (Sample)/COD (Positive Control).

### Sample collection

#### Pooled serum

As part of the national statutory brucellosis eradication scheme, serum samples are collected annually from all eligible animals (female bovines and entire bulls aged 12 months or over) in all cattle herds in Ireland. A sample of these herds was selected for the current study. Based on data available through the Animal Health Computer System (AHCS), stratified random sampling (based on two strata: ‘province’ and ‘herd size’) was used. There are four provinces in Ireland (Connaught, Leinster, Munster and Ulster). Two herd types were defined in this study – beef (containing >66% beef breed cows) and dairy (containing >66% dairy breed cows). The number of animals sampled was proportional to the number of herds with at least one birth registered in 2008 within each strata. In 2008, 87,396 herds had one or more births registered. Of these, 2,037 were mixed dairy and beef herds and were excluded. A further 30,894 herds were excluded because they were small (dairy herds <20 breeding cattle; beef < 10 breeding cattle). The proportion of the remaining 54,465 herds within each strata is shown in Table 1. The aim was to select a total sample size of 2,688 to allow for random selection of sufficient herds stratified on herd type (beef or dairy) and location (province) (Table 2). This sample size was based on herd-level Se and Sp (calculated from pooled-test Se and Sp values determined in the validation study) [19], a herd-level prevalence of 70% based on a previous study [16] and a participation rate of 50% for farms participating in the study during the 10 week collection period. Throughout the study period, animals were mostly kept on managed grassland. While 2,688 were initially contacted, 296 herds had to be excluded as they had either already completed their herd test prior to the proposed collection period or their contact details were inaccurate. Hence, 2392 herds were recruited of which 1659 were classified beef (containing <34% dairy breed cows) and 733 were dairy (>66% dairy breed cows), with approximately 204,000 animals. These farms represented just over 2% of all beef and dairy herds in Ireland. These study inclusion criteria, taking into account Irish national herd structure, would represent exclusion of less than 1% of dairy cows, but almost 15% of beef cows [20]. Permission for inclusion in the study was sought from all selected herdowners.

**Table 1:** Number of births registered, and number and percentage of herds where a birth was registered, during 2008, excluding mixed and small (dairy < 20 animals, beef < 10 animals) herds, by province and herd type

**Province**	**Animals**			**Herds**					
				**Number**			**%**		
	**Beef**	**Dairy**	**Total**	**Beef**	**Dairy**	**Total**	**Beef**	**Dairy**	**Total**
Connaught	264,141	51,439	315,580	12,316	1032	13,348	22.6	1.9	24.5
Leinster	300,933	254,292	555,225	9825	3908	13,733	18.0	7.2	25.2
Munster	304,081	621,957	926,038	11,077	10,174	21,251	20.3	18.7	39.0
Ulster	106,809	73,675	180,484	4752	1381	6133	8.7	2.5	11.3
Total	975,964	1,001,363	1,977,327	37,970	16,495	54,465	69.7	30.3	100.0

**Table 2:** Number of beef and dairy herds recruited within each province

**Province**	**Herd type**		**Total**
	**Beef**	**Dairy**	
Connaught	542	46	588
Leinster	434	175	609
Munster	474	449	923
Ulster	209	63	272
Total	1659	733	2392

Sample collection was conducted by private veterinary practitioners (PVPs) during a ten-week period from May to August 2009. The serum were placed in deep well blocks and stored frozen. Samples were later thawed, and a serum pool was generated for each herd, derived from up to 30 individual sera (each 10 μL). In herds containing <30 eligible animals, the pool included sera from all animals; in herds containing >30 eligible animals, 30 sera were randomly selected based on a randomisation list generated in Excel 2003. Sample pooling was carried out in Enfer Diagnostics, Naas, Co. Kildare, Ireland, an officially accredited laboratory. Testing of the serum pools was conducted at AFBI using the above-mentioned indirect ELISA.

#### Bulk milk

120 dairy herds were selected using convenience sampling for bulk milk analysis. Convenience sampling comprised contacting the owners of the first dairy herds from which serum samples were collected and requesting that they submit a bulk milk sample from their herd. Animals that contributed to the bulk milk sample included all lactating animals contributing milk for processing on the day of testing. In total, bulk milk was collected from approximately 20% of all dairy herds from which sera samples were obtained, within two weeks of serum collection. Using a prepared protocol, each herd-owner collected the bulk milk sample into a 20 mL universal container from an agitated bulk tank. Containers contained bronopol preservative tablets and were posted to AFBI for analysis using the above-mentioned indirect BVDV antibody ELISA test, for the purposes of comparison with results from the sample serum pools.

### Vaccine usage survey

A phone survey was used to clarify BVD vaccine usage in each of the study herds. Each study herd owner was contacted by phone by the first author, and an interview was conducted regarding duration and timing of vaccination and brand of vaccine used. Where relevant, at least three attempts were made to contact each study herd keeper. National usage data were obtained from sales data gathered by an industry survey [21]. GfK Kynetec is an international market research company. Using Microsoft Excel 2003, the data were summarised according to the number of vaccine doses sold by time of application (month), and by location (county; 26 in 4 provinces within the Republic of Ireland).

### Data analysis

#### Preliminary validation

The PP values from each of the 90 validation pools was recorded in a statistical software package (STATA®, Version 11.0/SE (Stata Corporation, Texas, USA, 2009) and subjected to Receiver Operator Characteristics (ROC) analysis, to determine the optimal cut-off PP to maximize Sp and Se of the test when used on pools [22]. Youden’s Index was calculated using the formula (Se + Sp-1) [23]. However, only values for Se and Sp of the indirect ELISA for BVDV, when the test is used on individual serum samples, are available (and not pool Se (PSe) and pool Sp (PSp)) (Manufacturer data on file).

#### Vaccine usage

Among the study herds, vaccine usage by county, seasonality and herd type were evaluated. Herds were classified as vaccinated within the previous 3 months, previous 6 months, previous year and non-vaccinated. Vaccine usage in study herds was compared to national usage data.

#### Seroprevalence study

All of the following analyses were conducted on non-vaccinated herds only.

### Pooled serum

The optical density result of each serum pool was recorded. The optimal cut-off PP value, as determined during preliminary validation, was used to classify each herd as seropositive or negative. Herd-level sensitivity (HSe) and herd-level specificity (HSp) values for the indirect ELISA test, when PCO was applied to a single pool of 30 samples from each herd, assuming a within-herd prevalence of 75% [3], were calculated using formulas derived previously [19]. Apparent (and true) herd-level prevalence was calculated from the proportion of positive pools using these HSe and HSp values. Associations between serum pool PP (quartiles) and herd type, herd size and province were tested using a chi-square test, while associations were also evaluated between herd-level seroprevalence classification (based on PCO) and the following parameters: herd type, province and herd size (quartiles) using a chi-square test.

ANOVA was conducted to determine associations between herd size and PP value of serum pools (quartiles). The goodness-of-fit of the models was assessed by examining residuals. Herd size was categorized into quartiles.

### Bulk milk

Bulk milk samples with PP ≥ 2.5 were deemed positive, as per manufacturer recommendations. The statistical software package: STATA®, Version 11.0/SE (Stata Corporation, Texas, USA, 2009) was used to calculate Cohen’s kappa coefficient, which is a measure of agreement between the bulk milk results and the seroprevalence results. Herds that had a different result in the bulk milk and seroprevalence studies were further investigated by whole herd analysis of individual serum samples to determine which classification was correct.

## Results

### Preliminary validation

**Figure 1:**
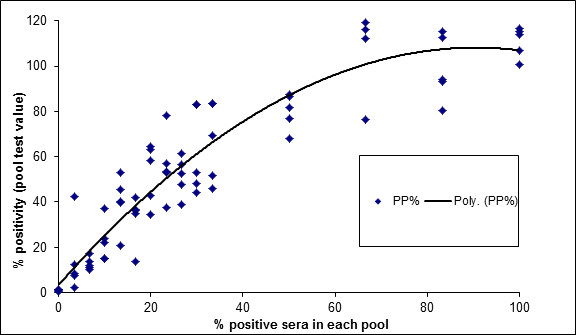
**Comparison between percentage positivity (PP) and percentage of positive samples, based on 90 validation pools.** A polynomial line of best fit is included.

There was good agreement between seropositive pools and seropositivity (R^2^ = 0.87; Figure 1), and a significant association between seropositivity and PP (p < 0.001). Based on a Receiver Operating Characteristic analysis of percentage positivity readings (PP), a cut-off PP of 2.12% resulted in a Se and Sp of 100% and 95%, respectively, relative to use of the ELISA on individual sera. A proposed cut-off (PCO) PP of 7.58% gave a relative Se and Sp of 98.57% and 100%, respectively. The PCO was chosen based on maximization of the prevalence independent criterion (Youden Index) in order for the ELISA to be used on bulk serum pools in the following seroprevalence study.

### Vaccine usage

Interviews were conducted with 1,113 (94.7% of the) study herd owners. In total, 214 (19.2%) of these herds had used BVD vaccine at some point, based on recall from the herd owner. 81% of these 214 were dairy herds, 72% had commenced vaccination programmes within the previous three years, 30% had been vaccinating for less than 2 years, and 14% had begun to vaccinate within the previous year.

**Figure 2:**
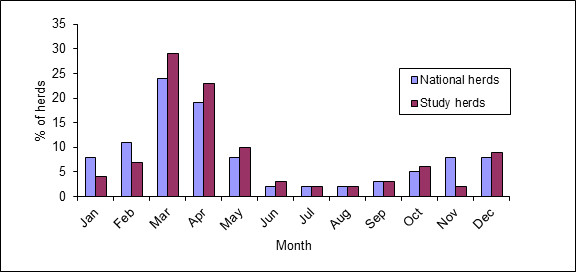
Monthly BVD vaccine usage on 1,113 Irish farms and nationally during 2009.

Approximately 840,000 doses of BVD vaccine were used in total by Irish farmers in 2009 [21], with a distinctly seasonal pattern of usage. Vaccination in this study showed a similar seasonal pattern (Figure 2) to overall vaccine usage in Ireland [21].

### Seroprevalence study

#### Pooled serum

Sera from 1,175 of the 2392 recruited herds were collected by 199 PVPs (approximately 61,000 sera from 60% of all bovine animals in these herds) during the study period. Participation was largely influenced by the decision of the herd-owner on the most suitable seasonal timing of their brucellosis herd test, with the herd owner of 1,217 herds choosing to delay until after the collection period, mainly for commercial reasons. 450 of the collected herds contained 30 or less samples. Using the PCO determined in the validation study, 13 herds were classified as seronegative. Excluding the 5.5% of herds where no vaccination history was available, apparent herd-level prevalence was calculated to be 98.9%. HSe and HSp were found to equate to PSe and PSp using the formulae derived previously [19]. Therefore true herd prevalence was calculated to be 98.7% (95% CI – 97.9-99.4%) in non-vaccinating herds.

Herd-level classification by province (herds with unknown vaccination history and vaccinating herds excluded) is outlined in Table 3. There was a significant difference between provinces for herd-level PP quartiles (p < 0.001), with highest PP values recorded in Leinster. Herd-level prevalence classification based on PCO did not differ significantly between dairy and beef herds (98.3% vs 98.8%, respectively, p = 0.595). ANOVA showed a significant association between herd size (quartiles) and herd-level PP quartiles (p = 0.02). However, no association was found on chi-square analysis of herd classification based on PCO, by either province (p = 0.366), herd type (p = 0.595) or herd size (quartiles) (p = 0.565).

**Table 3:** % of herds in each province by serum pool bovine viral diarrhoea (BVD) antibody ELISA Percentage Positivity (PP) result (presented as quartiles)

**Serum pool BVD antibody PP (%), in quartiles**	**% of herds**				
	**Connaught**	**Leinster**	**Munster**	**Ulster**	**Total**
0-58	24.1	16.5	49.1	10.3	25.0
58-74	21.4	25.4	42.0	11.2	25.0
74-89	25.7	27.4	35.8	11.1	25.3
89-135	23.1	36.7	29.0	11.3	24.7

#### Bulk milk

In total, 111 bulk milk samples were collected from herds, 41% of which were vaccinated. Herd-level prevalence in non-vaccinated herds was 92.3% (95% CI – 85.8-98.8%) based on results of bulk milk analysis. A comparison of the results from the pooled serum and bulk milk analyses of non-vaccinated herds, using the two different cut-offs, is presented in Table 4. Kappa analysis demonstrates 95.4% agreement between herd classification of seroprevalence based on pooled serum and bulk milk analysis when PCO is applied in non-vaccinated herds. Misclassification occurred with 3 non-vaccinating herds, using bulk milk analysis (at a cut-off of PP > = 2.5) compared to pooled serum (at a cut-off of PP = 7.58). In further evaluation conducted in 2 of the three above-mentioned herds based on an analysis of whole herd individual serum, both had been misclassified as negative using bulk milk analysis (two positive animals in a herd of 141, four positive animals in a herd of 42).

**Table 4:** Bovine viral diarrhoea (BVD) herd status for 111 herds in Ireland during 2009 (based on a comparison of bulk milk and pooled serum BVDV antibody results) in non-vaccinated herds

**BVD herd status based on bulk milk analysis (PP ≥ 2.5)**	**BVD herd status**			
	**(PP 2.12)**		**(PP 7.58)**	
	**Negative**	**Positive**	**Negative**	**Positive**
Negative	1	4	2	3
Positive	0	60	0	60
% agreement in classification	93.8%		95.4%	
(κ-value)	(0.32)		(0.55)	

The pooled serum results are separately presented at two different cut-off percentage positivity (PP) values (2.12 and 7.58). The level of agreement in BVD herd status, for non-vaccinated herds at each of the two cut-offs is presented, using kappa statistics.

## Discussion and conclusions

Apparent herd-level prevalence of BVD in non-vaccinating herds in Ireland was 98.7% where herds were classified positive when herd pool result exceeded PCO PP. National herd-level seroprevalence expressed as a percentage of positive herds depends on the cut-off selection, which in turn depends on within-herd prevalence. Assuming a test sensitivity and specificity of 100% and 98.2%, respectively, the true herd-level prevalence is 98.7% (95% CI:- 98.3-99.5%). Prevalence of BVD in this study is higher than the 70% individual animal-level seroprevalence estimated in previous studies [16]. However, assessment of animal-level BVD prevalence was not a goal of this study. Ireland is imminently embarking on an eradication programme and this study would yield useful information on levels of exposure to BVD to allow evaluation of progress. As this study is primarily concerned with determining herd-level prevalence due to disease exposure, including herd-level results from vaccinating herds could include herds where a positive antibody result was due to vaccination. Furthermore vaccinated herds were excluded due to factors that could have influenced the impact of vaccination on herd classification status. These include the variability of time between last vaccination and sampling on test result, the variable status of vaccination within vaccinating herds (part-herd vaccination, whole-herd vaccination), recall bias and the recognised impact of vaccination on bulk sample testing [6]. Prior to the present study, no information was available on herd-level prevalence of BVD in the Irish national herd, nor control measures implemented at farm level. Herd-level seroprevalence found in the present study is somewhat higher than in other countries for which published data are available [1,3,24].

The preliminary validation study was carried out to provide a proposed cut-off for use on serum pools of 30 samples. Two such cut-offs were found on ROC analysis of the results and the higher cut-off (PCO) was chosen based on maximization of the prevalence independent criterion (Youden Index) as a conservative approach to determining national herd-level seroprevalence. Additionally, PCO gave better agreement, in terms of herd classification, than using the alternative cut-off found on ROC analysis, when compared to results obtained in the bulk milk study (Table 4). Pools in the validation study were generated from a computer-generated simple random combination of known negatives and positives.

True herd-level seroprevalence of BVD can be determined from the number of seropositive pools [19]. The effects of pooling on Se and Sp of tests have been evaluated previously [19,25]. Epidemiological studies based on pool sample testing (PST) are not recommended on economic grounds if prevalence of disease is high [25]. However, PST will not give accurate assessment of within-herd prevalence and the only question that can be answered based on a single pooled sample per herd is whether the herd is infected or not. As herd classification is based on pooled tests in the seroprevalence study, estimation of herd-level Se and herd-level Sp is more complex because assumptions must be made about PSe and PSp. In this study it was assumed PSe = Se and PSp = Sp. It is likely that the PSe would be lower than Se especially when within-herd prevalence is low and pool size is large. The dilution effect on PSe will also be dependent on the exposed animal's concentration of antibody. In this study, one positive serum sample in the pool (equivalent to 3% within-pool prevalence) was used to determine cut-off in the ROC analysis. Furthermore, within-herd prevalence based on previous seroprevalence studies may be in the range of 60-85% [3]. Individual animals remain seropositive for a relatively long time after infection with maintained high levels of antibodies [26]. It is therefore contended that any effect of pool size on PSe would be mitigated by a combination of these factors. Conversely, PSp should exceed Sp [19] because dilution should make it less likely to have a false-positive pooled test result than a false-positive individual-test result.

Samples obtained from the last full national round of statutory blood test screening for *Brucella abortus* provided a unique opportunity for conducting this study in beef and dairy herds. It allowed an approach that would normally be prohibitively labour-intensive, expensive and dependent on issues arising from pooling sera. Sampling bias by PVPs refusing to participate was minimised by encouraging participation through clear communication of objectives to participating PVPs with a commitment to provide herd-level results. Procedures were implemented to ensure minimization of the effect of cross-contamination. Cross reaction with other agents is possible if a pool contained a viraemic animal. The prevalence of PI animals was not determined in this study. Cross-reacting agents could serve to lower the estimated prevalence. However, as the prevalence in this study is almost 100%, this factor is not envisaged as a major confounding factor in prevalence estimation. Furthermore, it has been suggested that pooling can reduce the impact of cross-reaction due to the dilution of cross-reacting agents with samples from non-infected animals [19]. Few publications exist on the use of ELISA tests on serum pools, though pools of 10 are widely used by the AFSSA, France [27]. Up to 30 animals in each herd were used in the serum pools in this study. A significant difference may have existed in certain herds, in terms of sampled animals. Bulk milk samples included all adult lactating females (except cows excluded due to illness, treatment etc.) while serum pool analysis (98.2%) comprised 30 randomly selected animals from all female animals and breeding bulls >1 year from the same herds. The impact of the difference in sampled animals was not investigated in detail in this study. Kappa analysis demonstrated 95.4% agreement between classification of non-vaccinating herds based on bulk milk (92.3% positive) and classification based on serum pool analysis (97.0% positive) when PCO was applied. Performance characteristics of the test to detect antibodies in pooled sera were demonstrated to exceed those in bulk milk by whole herd analysis of individual animals carried out on those herds misclassified as negative using the bulk milk test compared to the analysis of pooled serum samples. However, only two such herds were evaluated in this study. Bulk milk, though widely used in seroprevalence studies, limits the application to dairy herds, and lowered sensitivity of the ELISA has been identified in the application of bulk milk analysis [28,29]. Bulk milk based seroprevalence studies may also be influenced by timing in seasonal calving populations and herd average yield [30], although this is considered a minor issue. It is important to emphasise that the validation of the ELISA test on serum pools was performed against the same ELISA used on individual samples. While this is not optimal, as the Serum Neutralisation test is the accepted gold standard test for validation, large quantities of serum with known SNT readings were not available, as many laboratories no longer routinely carry out this test for a variety of reasons [31]. However, the ELISA has been validated by the manufacturers against the SNT when used on individual sera with a Se and Sp of 100% and 98.2%, respectively. Larger-scale work is advised on the use of serum pools with low seroprevalence to confirm the cut-off found in this study and to more accurately validate the use of this test on serum pools. While desirable for accurate estimation of national seroprevalence, analysis of all samples individually would obviously be prohibitively expensive.

The distinctly seasonal usage of BVD vaccine reflects the calving pattern of the Irish herd, suggesting that most vaccine is used prior to breeding as per manufacturers’ recommendations. The majority of vaccine is used in dairy herds (38% versus 9% of beef herds), despite seroprevalence being equivalent in both herd types. This suggests a significant information deficit in the beef sector. Recall bias could have arisen in this study among farmers surveyed. Vaccine brand used was determined to minimize recall bias and confusion with vaccines used to control other diseases.

Herd size was determined to be a significant risk factor with regard to pool PP (quartiles), however, no association was found between this parameter and herd classification based on PCO. This is likely to be because herd-level prevalence is almost 100%, which precludes differentiation between positive and negative herds. Excluding herds where no vaccination history was available, herd-level prevalence was 98.7% (95% CI – 97.9-99.4%) in non-vaccinating herds. While vaccination affected individual animal prevalence in a previous study, a lack of a significant effect of vaccination on herd-level prevalence was also found in that study [32]. Herd level prevalence was only calculated in non-vaccinating herds in this study. An association between herd-level BVDV seroprevalence and herd size is in line with previous findings [3], however, this may be a confounded risk factor [33].

BVD is widely recognized as a cause of a range of conditions affecting a number of organ systems with the outcome depending on immunocompetence [8]. Economic losses from BVD infection have been reviewed previously [34,35]. With a cattle population in Ireland comprising 1.087 million dairy cows and 1.105 million beef cows as well as growing animals [36]), and with numbers of farms falling by 3-4% per year as average farm size increases, significant disease control challenges lie ahead. Current BVD controls have, until recently been limited almost exclusively to vaccination, a strategy which is unlikely to have a significant impact on national herd-level prevalence [14]. Additional measures such as identification and elimination of PI animals, certification of herds and implementation of biosecurity measures are required to make any impact on herd-level seroprevalence in Ireland considering the current high levels. In addition to those EU member states already officially free of disease, other countries (Netherlands, Belgium, France, Germany, U.K., Spain and Italy among others) are at various stages of herd certification/eradication [27,37-40]. Knowledge of BVD seroprevalence is necessary for designing and implementing effective concerted national control and eradication measures. Animal Health Ireland (AHI), an industry body charged with the national leadership and coordination of production disease issues in Ireland, is currently assembling information with a view to informing policy on their control. AHI has implemented a voluntary scheme of PI elimination for calves born in Ireland since January 2012. This approach is planned to become mandatory, through legislative change, for all calves born in Ireland from January 1^st^ 2013. Furthermore, recent efforts by AHI and other agencies to increase awareness of this disease and encourage implementation of cost-effective controls, including screening, elimination of PIs, vaccination and biosecure practices will help reduce prevalence. However, ultimately these must be coupled with a herd accreditation programme and effective implementation of statutory measures to reduce national prevalence.

## Abbreviations

AFBI = Agri-Food and Biosciences Institute, Stormont, Belfast, Northern Ireland; AHI = Animal Health Ireland; ANOVA = Analysis of variance; BVDV = Bovine virus diarrhoea virus; CI = Confidence Interval; COD = Corrected Optical Density - The value of each sample and reference sample was obtained by subtraction of the OD value of each control antigen-coated well from that of the parallel viral antigen-coated well in the ELISA; EU = European Union; ELISA = Enzyme-linked immunosorbent assay; κ = Cohen’s kappa coefficient; μL = Microlitre; OD = Optical Density - The absorbance of each sample well at 450 nm was measured on a microplate plate reader; PCO = Proposed cut-off; PP = Percentage Positivity; PVPs = Private Veterinary Practitioners; ROC = Receiver Operator Characteristics analysis; Se = Sensitivity; SNT = Serum Neutralisation test; Sp = Specificity.

## Competing interests

The corresponding author is an employee of MSD Animal Health (Ireland), however, the company played no role in study design, data collection and analysis, decision to publish, or preparation of the manuscript. The remaining author(s) declare that they have no competing interests.

## Authors’ contributions

DJBC conceived and designed the study, organised the blood sample collection and analysis, designed and conducted the owner questionnaires, collected vaccine usage and production data and drafted the manuscript. SJM and TAC participated in the design and coordination of the study, and DJBC and TAC performed the statistical analysis. SJM and MLD helped to draft the manuscript. All authors read and approved the final manuscript.

## Authors’ information

DJBC is a Bachelor of Veterinary Medicine and works as Technical Manager, MSD Animal Health (Ireland). TAC works as principal statistician in the Centre of Veterinary Epidemiology and Risk Analysis (CVERA), within the School of Agriculture, Food Science and Veterinary Medicine at University College Dublin. MLD is UCD Professor of Veterinary Clinical Studies. SJM is UCD Professor of Veterinary Epidemiology and Risk Analysis and CVERA Director.
